# Biomimetic heterogenous elastic tissue development

**DOI:** 10.1038/s41536-017-0021-4

**Published:** 2017-06-08

**Authors:** Kai Jen Tsai, Simon Dixon, Luke Richard Hale, Arnold Darbyshire, Daniel Martin, Achala de Mel

**Affiliations:** 1 Division of Surgery and Interventional Science, University College London, London, UK; 2 Biomer Technology Ltd, Runcorn, UK; 3 The Centre for Altitude Space and Extreme Environment Medicine, Univeristy College London, London, UK

## Abstract

There is an unmet need for artificial tissue to address current limitations with donor organs and problems with donor site morbidity. Despite the success with sophisticated tissue engineering endeavours, which employ cells as building blocks, they are limited to dedicated labs suitable for cell culture, with associated high costs and long tissue maturation times before available for clinical use. Direct 3D printing presents rapid, bespoke, acellular solutions for skull and bone repair or replacement, and can potentially address the need for elastic tissue, which is a major constituent of smooth muscle, cartilage, ligaments and connective tissue that support organs. Thermoplastic polyurethanes are one of the most versatile elastomeric polymers. Their segmented block copolymeric nature, comprising of hard and soft segments allows for an almost limitless potential to control physical properties and mechanical behaviour. Here we show direct 3D printing of biocompatible thermoplastic polyurethanes with Fused Deposition Modelling, with a view to presenting cell independent in-situ tissue substitutes. This method can expeditiously and economically produce heterogenous, biomimetic elastic tissue substitutes with controlled porosity to potentially facilitate vascularisation. The flexibility of this application is shown here with tubular constructs as exemplars. We demonstrate how these 3D printed constructs can be post-processed to incorporate bioactive molecules. This efficacious strategy, when combined with the privileges of digital healthcare, can be used to produce bespoke elastic tissue substitutes in-situ, independent of extensive cell culture and may be developed as a point-of-care therapy approach.

## 3D Printing Artificial Elastic Tissues

Solvent-free thermoplastic polyurethanes (TPU) could be used to 3D-print artificial tissues saving time and money. Achala de Mel and colleagues at University College London used open-source 3D-modelling software and commercially available 3D printers to fabricate a bespoke tracheal stent from custom-made TPU. The team was able to control the material’s porosity with 3D-design, which could facilitate its vascularisation if implanted. The trachea was mechanically and structurally similar to that of an adult, showing longitudinal elasticity and radial rigidity. When attached to a ventilator system, it responded well to pressures similar to those of inspiration, forced expiration, coughing or crying. 3D-printed trachea was treated with bioactive molecules so cells could potentially adhere to and proliferate on its surface. This method could be used to fabricate bespoke elastic tissue substitutes, avoiding costly and time-consuming cell-culture techniques.

## Introduction

The long-standing desire to repair or replace, damaged or diseased organs,^1–6^ was reflected in ancient mythology, biblical stories and in fiction, before it evolved into a scientific and clinical plausibility (Supplementary Figure 1). Tissue engineering methodologies^7–9^ could be optimised for emergency surgeries and for routine use in remote parts of the world that have no access to specialised laboratories. Additive manufacturing, 3D printing and associated multidisciplinary technologies present incredible opportunities for developing bespoke prostheses, with flexible design capabilities and greater geometric accuracy for tissue engineering.^2^ Fused deposition modelling (FDM) is one of the most widely used 3D printing techniques. This methodology for building 3D structure can facilitate material anisotropy, which is an attractive feature when aiming for hetergenous tissue biomimicry. A range of thermoplastic polymers including Thermoplastic polyurethanes (TPU) have been demonstrated as being 3D printable using FDM,^10, 11^ as well as the more commonly used polycaprolactone (PCL) and polylactic acid (PLA.^12, 13^) although PLA and PCL are inherently relatively stiff materials, and as a consequence their application is limited—materials that offer greater flexibility, and tailorability to the application at hand such as TPU are desired.

Commercally available FDM printers are relatively economical, user friendly and do not require the specialised staff and safety considerations associated with processes such as selective laser sintering. The techniques demonstrated here are therefore widely accessible and applicable even with limited resources.Printing flexible filaments by FDM is not without challenges, such as directing filaments towards the extruder (Supplementary Figure 2), requires manual adjustments to almost all currently available FDMs. Only a small number of studies have explored direct printing of ‘solvent free’ soft elastomers, which is a limitation associated with ‘indirect printing’, where solvent based elastomers are introduced to a 3D sacrificial mould,^14^ which is an additional step that is not required for direct 3D printing. With indirect printing, the implementation of an internal pore structure might limit the thickness and dimensions of the resulting 3D structure due to the limited ability for solvent based elastomers to homogenously penetrate through the patterned “mould”. In addition, direct printing allows a combination of mechanical properties from the use of multimaterials to generate a tailored heterogeneous scaffold.

Despite the theoretical ability to produce a limitless range of TPU elastomers, practical and commercial considerations dictate that in reality only a small percentage of the potential material grades are commercially available. In the case of medical device applications the percentage is further limited by the use of additives and processing aids in the polymer manufacturing, which affect both biocompatibility and function. This is evidenced by the very limited number of new medical grades of TPU entering the market over the last decade. Here we have custom synthesised a polyester (polycaprolactone) polyol based (TPU 80) and a polyether polyol based (TPU 90) formulation (Supplementary Figure 3) in a ‘designed in*’* rather than ‘engineered out*’* approach to demonstrate the unique opportunities with TPU in developing tissue substitutes. Both polymers were synthesised using a precisely controlled step addition (prepolymer approach) process using an aromatic diisocyanate and a short linear diol chain extender, which were subsequently extruded into 1.75 mm filaments and then 3D printed. We used two different types of commercially available FDM printers to evaluate the efficacy of fabrication of TPU filaments. The models used for 3D printing were designed and sliced to incorporate micropatterns using open source software, Blender^™^ and slic3r^™^, respectively.

## Results

### Structural and mechanical biomimetic design flexibility with direct 3D printed thermoplastic polyurethane

**Fig. 1:**
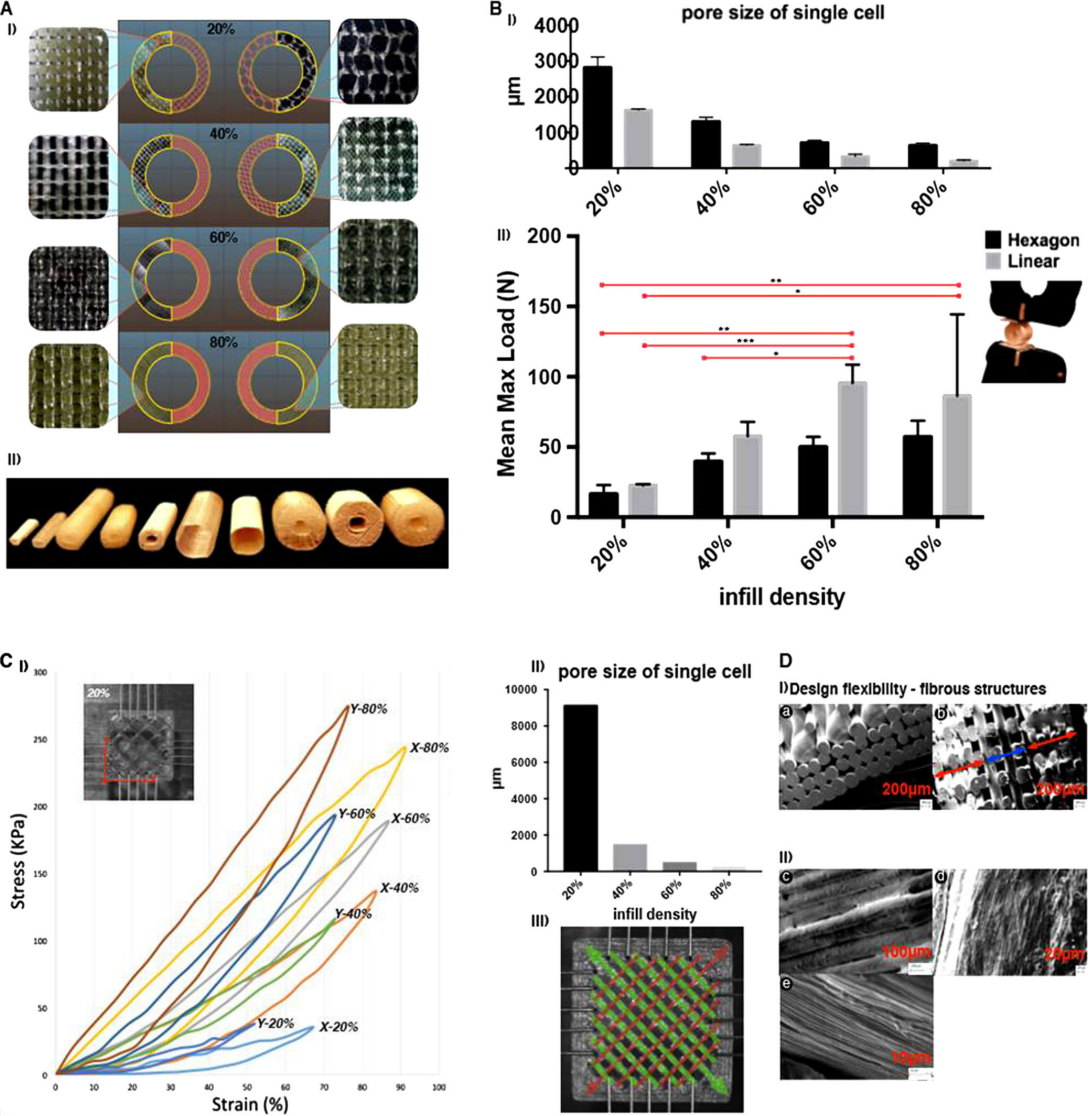
Architectural and mechanical biomimetic design flexibility with direct 3D printed thermoplastic polyurethane. **a** (i). 3D hollow tubular CAD (20 mm height × 15 mm diameter) was sliced (Slic3r) to have a range of infill densities in two different infill patterns (hexagonal and linear). Corresponding 3D printed structures were obtained with TPU90 with clear morphological definition. (*n* = 6) (ii). A range of 3D printed tubular structures were obtained by varying the basic code (indicated in supplementary methods) for (the wall thickness, infill density and diameter) of the tubular hollow CAD structure for **a** (i). **b** (i). The ‘pore’ size is significantly greater in the hexagonal constructs than linear. The increase in infill density decreased ‘pore’ size, but an exception observed between 60 to 80% in the hexagonal infill (*P* > 0.05). (ii). Compression strength of 3D printed structures corresponding to morphological details described in **a** (i). The hexagonal infill was significantly more compliant than the linear infill at 40, 60, 80% (*P* < 0.001) but not 20% (*P* > 0.05). Overall the linear infill requires significantly more force to compress (*P* < 0.05). **c** (i) The surface architecture of 3D printed TPU90 scaffolds (15 × 15 × 1.5 mm) with significantly different fibre thickness in opposite directions. (ii) Biaxial test demonstrating a density dependant increase in elastic modulus of scaffolds, as well as material anisotropy with significantly higher compressive stress in *y*-axis, and greater elongation in *x*-axis. (*p* < 0.05). (iii) The pore size of the scaffolds is inversely related to the infill density (*p* < 0.05). **d** (i) **a** SEM of a 3D printed TPU90 as a ligament structure that mimics collagen fibre morphology **b** with an infill gradient (indicated with *double headed arrows*) to modulate varying tissue interphases. (ii) SEM of Porolay (PVA + TPU) biomimicking fibrous morphology as potential substitutes for connective tissue

We have 3D printed tubular structures (Supplementary Figure 4) and have evaluated the definition and the structural integrity of prints. (Fig. 1a) The surface architecture of 3D tubular scaffolds, printed with TPU90, demonstrated design flexibility and definition, with the surface area of a pore of a given pattern found to be inversely proportional to the infill density. (Fig. 1a and b) The hexagonal style infill produced a more compliant structure compared to a linear infill of the same density without compromise to pore quality (Fig. 1b). We demonstrated the rapid adaptability to mimic a range of elastic tubular structures (Fig. 1a and b, Supplementary Figure 5) and we also demonstrated the possibility to create pores distributed throughout all planes (Supplementary Figure 4 E-I) which would be attractive for vascular ingrowth. We demonstrated simple material anisotropy dependant on infill pattern with biaxial test TPU printed structures (*n* = 5) (Supplementary Figure 6, Fig. 1c i, ii, iii). The scaffold is significantly (*P* < 0.05) more flexible in *x*-axis and it is more rigid in *y*-axis. It also demonstrated an infill density-dependant increase in elastic modulus. We also explored a commercially available TPU (Porolay) which is available as a co-extrusion with Poly(vinyl alcohol) (PVA). The PVA can be dissolved away in water conferring porosity and flexibility on the final print. Here we demonstrate biomimicry of a ligament structure with distinct collagen bundles at the microscopic level (Fig. 1d i,ii).

### Surface modification for biofunctionalisation; post processing of 3D printed scaffolds

**Fig. 2:**
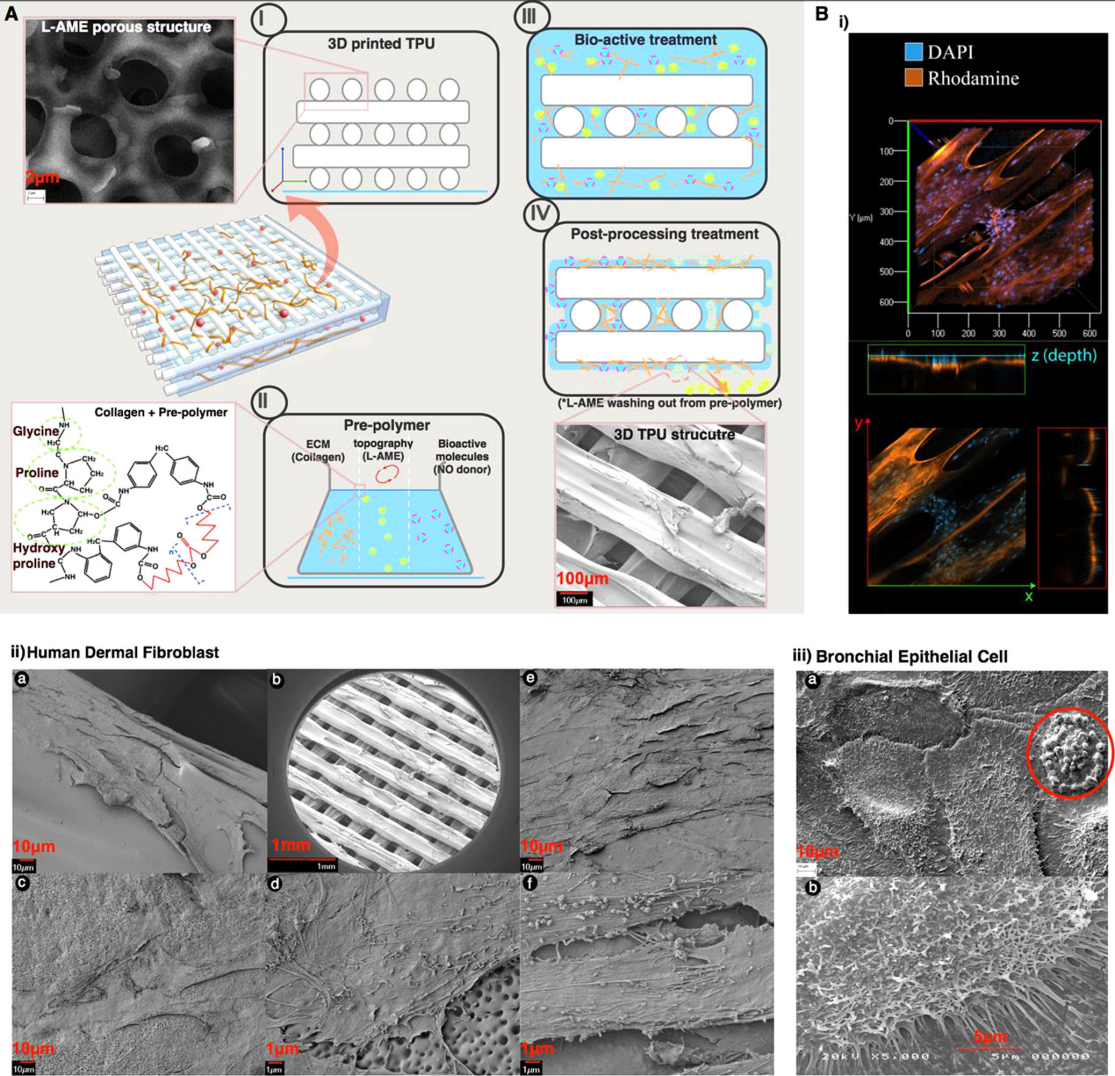
Surface modification for biofunctionalisation; post processing of 3D printed scaffolds. **a** Diagrammatic illustration of post processing of 3D printed scaffold, with collagen as an example of ECM component, L-AME as example bioactive molecule, which were introduced with solution based pre-polymer acting as a ‘glue’. Subpanels i-iv indicate the order in which post-processing of scaffolds can be carried out to obtain a biofunctionalised 3D printed scaffold. **b** Cell interactions on 3D printed scaffold surface (i) DAPI Fluorescence staining (Blue) demonstrating presence of cells on the scaffold (stained in red). SEM images. (ii) *a*, *b*. (higher mag). HDF at day 3 on 3D printed TPU90 surface. *c*, *d*. (higher mag) HDF at day 3 seeded on 3D printed TPU90 surface functionalised with L-AME. *Red arrow* indicating L-AME pores on the scaffold. *e*, *f*. (higher mag) HDFs at day 3 seeded on 3D printed TPU90 surface functionalised with collagen. (iii) BEC (at day 14) seeded as a submerged culture on 3D printed TPU80 surface biofunctionalised with collagen and L-AME. a. Mucus synthesis indicated within *red circle*. b. a cell prominent with Microvilli

Surface functionalisation can modulate cell-material interactions, including rapid cell adhesion and proliferation on a scaffold. We demonstrate a post processing method, which enables surface bio-functionalisation for enhanced cell-adhesion, whilst maintaining bulk mechanical properties and structural integrity of 3D printed constructs. To demonstrate this in-vitro, we have used a solution-based TPU pre-polymer system which we have hybridised with collagen, as an example of extracellular matrix (ECM) component and L-Arginine methyl ester (L-AME) as a porogen^15^ (Fig. 2a, Supplementary Figure 7). We have tested the viability and interactions of human dermal fibroblasts (HDF) and bronchial epithelial cells (BEC) (Fig. 2b). Quantification with alamar blue viablity tests (*n* = 8) has demonstrated that the L-AME and collagen combined pre-polymer treated surface has the highest cell viability (*p* < 0.05) at 24 and 72 h for HDF and BEC, respectively (Supplementary Figure 8).

### Structural, mechanical biomimicry of heterogenous tissue; trachea as an exemplar

**Fig. 3:**
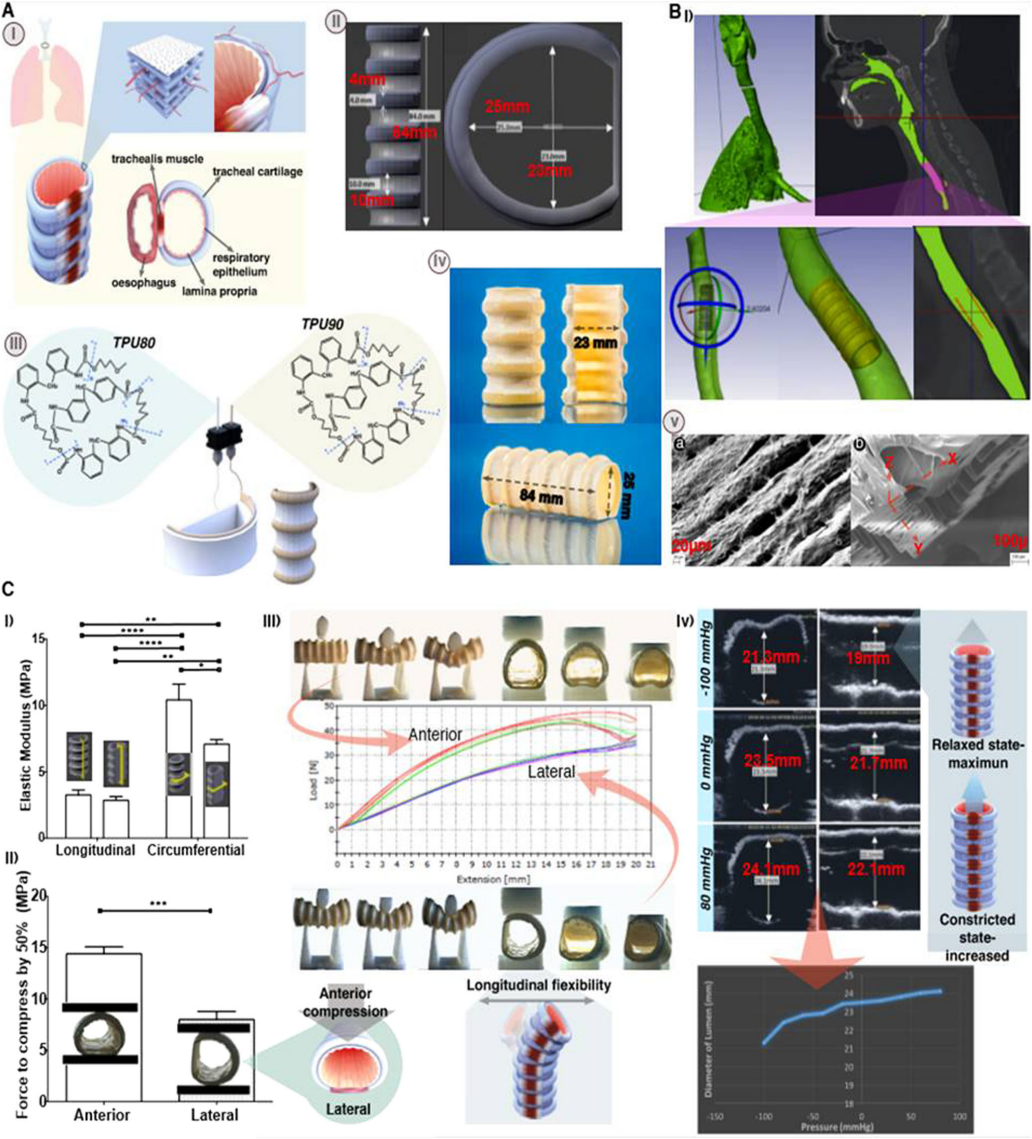
Structural, mechanical biomimicry of heterogenous tissue; trachea as an exemplar. **a** Structural biomimicry of an adult trachea: (i) Trachea is relatively static, longitudinally flexible but radially rigid with intermittent cartilage ‘c’ shaped rings, which maintains luminal structure, with a softer trachealis muscle in the posterior providing the compliance for optimal ventilation.^13, 18, 19, 24–26^ (ii) 3D CAD model of trachea generated using Blender^™^ software. (iii) STL of the CAD (ii) was sliced in slic3r^™^ (SI) and 3D printed with a dual extrusion FDM printer with TPU 90 to mimic cartilaginous rings and TPU 80 for softer trachaelis muscle and connective tissue. (iv) 3D printed TPU biomimetic tracheal structure. (v) Ultrastructure of the 3D printed biomimetic tracheal construct; SEM images of respective cross sectional and surface images. **a** luminal surface post processed with L-AME and collagen **b** cross sectional view illustrating the structure to potentially facilitate blood vessel infiltration. **b** (i) Simpleware^TM^ software modelling of generic CAD of a trachea to match a patient trachea (CT scan) as a potential stent. **c** Mechanical and functional biomimicry. (i) Tracheal segements (*n* = 12) demonstrated greater strength radially (*p* < 0.01) and greater longitudinal elongation (*p* < 0.05) resulting in significantly lower, elastic modulus (*p* < 0.05) for longitudinal segments (*n* = 12) compared to circumferential segments (*n* = 10) with no significant difference of elastic modulus between anterior and posterior longitudinal segments (*p* > 0.05) but significantly higher elastic modulus for anterior circumferential segments compared to that of posterial circumferential segments. (ii) Greater force (*p* = 0.05) was required to compress tracheal segments (25/23) (*n* = 8) with anterior compression than required to compress laterally. (iii) Flexibility in bending; the constructs were bent in the anterior plane with ease without causing luminal closure and returned to their original shape upon removal of the force. Constructs (*n* = 5) were laterally bent with even greater ease (*p* = 0.05). (iv) Compliance under a range of pressures monitored with ultrasoundscans, with the probe placed **a** across and **b** along the 3D printed tracheal construct. *Double headed arrows* on scans indicate maximum distension at a given pressure

To demonstrate applications of our in-situ elastic tissue mimicry approach and design flexibility, we present evidence for structural and mechanical biomimicry of an adult trachea. (Fig. 3a-i). Dual printing of TPU has enabled complete, structural biomimicry of this heterogenous tissue construct. The cartilage rings were printed with biostable TPU 90 and trachealis muscle and intermediate supporting tissue was printed with TPU 80 (Supplementary Figure 9, Fig. 3a-ii, iii, iv). As a proof of principle, we have post-processed the tracheal constructs to incorporate mesoporous luminal surface whilst retaining bulk mechanical properties and porosity (Fig. 3a-v) to support potential angiogenesis once implanted in the body. This adult size construct (84 × 25 × 23 mm) of a complete tracheal construct was printed and processed within 6 h. Such rapid efficiency is not possible with any other technique presently available for tissue mimicry with current processes requiring more typically a minimum of 24 h to complete. Here we also show a computational attempt to adapt a generic adult tracheal stent to be patient-specific with DICOM data of a CT scan (Fig. 3b). This proposed approach of in-situ printing can be coordinated remotely with centres to offer bespoke, point of care therapy. The construct demonstrated good handling when sutured with a 4.0 proline suture to a porcine respiratory system (Supplementary Figure 10) and air tightness when air flow was introduced through an air bag with the trachea-lung system placed under a saline bath. We demonstrate the mechanical biomimicry^16–21^ and tissue anisotropy of the structure (Fig. 3c) by excising distinct components (Supplementary Figure 11) to perform tensile tests that confirmed tissue anisotropy that is expected of tracheal tissue, with longitudinal elasticity (3.25 MPa anterior and 2.85 MPa posterior segments) and radial rigidity (10.42 MPa anterior, 7.08 MPa with posterior segments) (Fig. 3c-i) In addition,we tested its ability to bend under physiological conditions without luminal closure and regain its form upon removal of force, which correlates well with the compression tests with 12.86 MPa for lateral compression and 15.86 MPa for anterior compression (Fig. 3c ii, iii). We have explored the degree of biomimicry of the tracheal construct under a range of pressures with the trachea attached to a ventilator system to determine potential response during coughing, crying and forced expiration and inspiration with ultrasound measurements^22^ (Supplementary Figure 12). The structure has sufficient strength to prevent luminal collapse but is not too rigid to compromise optimal ventilation. The 3D printed construct demonstrated an average of 15% change in diameter when subjected to a pressure change from −100 to +80 mmHg (Fig. 3c iv, Supplementary Information). Furthermore the tests have shown ultrasound to be informative in measuring compliance and this technique can be adapted to investigate the functionality of a range of 3D printed elastic structures. We demonstrated that by simply changing the ratio of luminal diameter from 25/23 to 17/25 (Supplementary Figure 13) whilst maintaining all other parameters constant, we can achieve significantly different (*p* < 0.05) mechanical strength for both lateral (14.86 MPa) and anterior compression, (45.71 MPa). This suggests the potential for permutations that one could test to fine tune a structure of interest, including the structural-mechanical nuances within the cartilage rings through the introduction of design variations within the ring structure.

## Discussion

A tubular elastic structure with varying diameter and thickness is a common structural feature of many organs^23^ and here we have demonstrated the flexibility and adaptability of direct 3D printing of TPU to structurally and mechanically mimic these structures. The ability to modulate infill patterns with relatively simple changes to computer assisted designs can influence the compliance properties of the resulting 3D printed structures, enabling mechanical biomimicry and controlled porosity that could facilitate potential therapeutic angiogenesis. This channel to fine tune artificial elastic tissues by directly printing with TPU offers excellent opportunities for a plethora of elastic tissue replacements. The trachea is an outstanding candidate to demonstrate structural heterogeneity, material anisotropy and biomechanical versatility^13, 18–21, 24–26^ and we have successfully demonstrated a structural and mechanical biomimicry of a full structure of an adult trachea, particularly with distinct cartilage rings and trachealis muscle as an exemplar biological structure.

Previous studies have shown encouraging results with TPU as a relatively small “patch” in-vivo.^11^ Although there was still a need to produce a scaffold, which can essentially self-support in form and function, which we have addressed here, with dual printing of TPU for the first time without a solvent based TPU printing system, that mimics to a great extend the biomechanics and macro structure of an adult trachea, (Fig. 3) thus supporting our aim to develop a path with 3D printing TPU for wider clinical applications. A solvent free, biogedradable, water based TPU has also been tested^10^ as a scaffold, but is not self supporting, and required to be printed with a bespoke, highly complex system which would be less practical as a potential point-of-care system. Porolay, with its combination of PVA and TPU as demonstrated here in this study, offers yet another interesting co-extrusion polymer system with a mode of controlling structural properties of TPU based 3D structures.

Cell-material interactions can be influenced by the presence of bioactive molecules such as those that mimic ECM for cell adhesion, antibacterial molecules to prevent implant associated infection, and antithrombogenic molecules to confer haemocompatibility and mimic the endothelium.^15, 27–29^ This surface modification method demonstrated here can serve as an effective platform technology for the integration of a plethora of biomolecules for cell-material interactions. It is also an effective ‘seal’ at the luminal phase to modulate permeability. The solution based pre-polymer system, which acts as a ‘glue’ is an alternative to genipin, muscle adhesive proteins, which may be used as a cross linker for surface functionalisation and molecular biomimicry. 3D printed TPU elastic structure mimics should be ideally introduced in-vivo with autologous platelet rich plasma (PRP) gel as routine practice to accelerate blood vessel formation. PRP gel consists of growth factors that promote angiogenesis, as well as stem cell migration to induce wound healing^30–32^ Therefore PRP gel may work in synergy with optimally-interconnected porous networks which 3D printing can create to facilitate vascularisation of structures. It is of interest for future studies to explore 3D printing of TPU to produce celia mimics and potentially replace a role for celiated epithelium within the lumen as demonstrated previously with 3D printed microfibers for other applications.^33^

The method of TPU fabrication and biofunctionalisation presented here may be of interest for the rapid generation of experimental models to explore mechanosensitivity of cells.^34, 35^ There is also further potential to optimise FDM printers for highly flexible TPUs to eliminate the need for routine manual adjustments during processing, potentially through advanced FDM printers, which can directly introduce polymer pellets free from solvent thus bypassing any limitations associated with filaments. The innovative platform technology which we present in this study is elegantly simple, using minimum materials, which can be maintained as stock extrusions that can be processed on standard, accessible printing equipment without the requirement for extensive preparation or post process clean up. Regulatory qualification of tissue implants are both device and location specific, following closely controlled national and internationally recognised guidelines and standards. By reducing the number of material components and process steps this flexible, core process can lend itself to multiple device applications and constructs, which are easily sterilisable within an operating theatre setting and would enable this to potentially progress to become routine practice in the clinical setting at the point of care.

## Methods and materials

### Materials for biomimetic scaffolds and characterisation

Thermoplastic polyurethanes, with the compositional data indicated in the Table 1, were synthesised using a proprietary two stage process involving the preparation of a controlled prepolymer of polyol with diisocyanate and a subsequent chain extension step with a short chain diol to form a highly linear, narrow molecular weight distribution TPU polymer.

**Table 1:** Compositional data of thermoplastic polyurethanes

Polymer code	TPU 90	
Description	Linear aromatic polyether urethane	
Raw materials	%	Supplier
Polyether polyol (2000 mwt)	63.01	IMCD
4,4 -Methylenebis(phenyl isocyanate),	29.64	Borsodchem
1,4 Butane diol	7.35	Whyte chemicals
	TPU 80	
Polycaprolactone diol (2000 mwt)	68.76	Perstorp
4,4-Methylenebis(phenyl isocyanate),	25.72	Borsodchem
1,4 Butane diol	5.52	Whyte chemicals

#### Filament formation

1.75 mm filaments were extruded for each of the above TPU’s using a standard plastic extrusion set up as illustrated in Supplementary Figure 14

The polymers were dried at 80 °C for a minimum of 4 h prior to extrusion.

We also used commercially available Porolay, LAY-FOMM40 and LAY-FOMM60, filaments to demonstrate the versatility of TPU that could potentially be utilised to mimic elastic tissues.

### CAD and 3D printing

Open source 3D modelling programme Blender^™^ (Stichting Blender Foundation, Amsterdam, Netherlands) was used to model 3D objects to obtain STL file formats, which were then sliced in open source Slic3r^™^ software to obtain G-codes to be printed with Sharbot Next generation desktop 3D printer (Sharebot S.r.L, nibionno (lc) Italy) or Makerbot slicing software (MakerBot^®^ Industries, LLC, USA) to convert STL into.×3 g for printing using 3dison Mulit 3D printer (Rokit, Korea). Both printers have dual extrusion capability, and this feature was utilised to print tracheal constructs with both TPU 90 and TPU80 for the respective tissue components. Filaments were extruded at a temperature of 236 °C at a rate of 2 mm/s.

We also used a 3D image processing and mesh generating software, Simpleware^™^ (ScanIP and + CAD) to obtain and generate pores within 3D scaffolds, as well as density gradients for potentially mimicking distinct tissue interphases. (Figure Supplementay Information)

We also used ScanIP and + CAD to explore the potential to fit a generic adult tracheal stent to be a bespoke structure. (Fig. 2b ii)

### Biomechanical testing

A series of biomechanical tests were performed on test samples (*n* = 8) using Instron 5565 with ‘Bluehill’ software and material testing frame with a 500 N capacitor. The sample type and test settings for the respective tests are shown in the following Table 2.

**Table 2:** Details of sample types and test settings of respective biomechnical evaluations

Test	Tensile	Compression	3 point bend
Sample type	Dumbell section (50 mm length) excised as indicated (Supplementary Figure 10)	Samples of approx 15 mm in height	Adult trachea samples 84 × 25 × 23 mm
Test Conditions	Cross head speed −20 mm/min Guage length-20 mm	Crosshead speed 10 mm/min	Crosshead speed-10 mm/min
Test description summary	Tensile tests were carried out essentially in accordance with ISO37:2011	Samples were compressed to 50% of their original width. Each sample was pre-conditioned (at a rate of 15 m m/s)	Trachea samples were subjected to three point bend test and the force required to move at a speed of 10 mm/min was recorded

### Biaxial test

TPU90 was 3D printed obtain 15 × 15 × 1 mm square scaffolds with rectilinear infill 20, 40, 60, and 80% densities. Samples were immersed in a water bath, which was maintained at 37 °C. Each sample was pre-conditioned at 100 mN with 8 cycles within 10 s, followed by 100% of displacement dependant tests within 40 s (BioTester Biaxial test system, CellScale, USA).

### Compliance testing of tracheal constructs

Each end of the tracheal tests samples were sealed with an intubation tube with an endotracheal cuff inflator. Attached to one tube is a pressure monitor measuring mmHg (Comark, Fluke, UK) and to the other a 50 ml syringe. This system can then manually change the pressure within the 3D printed tracheal construct from −200 to +100 mmhg. The centre of the trachea was placed with an VFX 9–4 ultrasound probe (Sonoline Antares, Siemens Medical Solutions Inc, USA) set to a frequency range of 3.8 Mhz with tissue harmonics (THI) engaged and a dynamic range of 55db. (Supplementary Figure 4). This generated the optimal image of the trachea both longitudinally and radially. The diameter of the trachea was then traced within a range of induced pressures. An Oxylog 3000 ventilator (Dräger, UK) was also used to provide the pressure instead of a syringe on a number of tests. Testing a range of pressures from 15 to 60 mBar at a frequency of 20 breaths/minute. An inflatable balloon was attached to the opposite end in place of the pressure monitor. This contained the high volume of air generated by the ventilator in order for it to function.

Surface functionalisation

- Solution-based TPU pre-polymer system Poly(hexamethylenecarbonate) diol, 2000 mwt, (UBE, industries Ltd) was placed in a 250 ml reaction flask equipped with mechanical stirrer and nitrogen inlet. Flake 4,4′-Methylenebis(phenyl isocyanate), MDI, (Sigma Aldrich), was added to the polyol and then reacted, under nitrogen, at 75–85 °C for 90 min to form a pre-polymer with an NCO content of 4%. Dry Tetrahydrofuran (Sigma Aldrich) was added slowly to form a 20% solution of pre-polymer by weight.
- Biofuctionalisation Exemplar bioactive molecules, collagen (BioHorein, Thaiwan) and L-arginine methyl ester dihydrochloride (L-AME) (Sigma Aldrich) were dispersed within the pre-polymer and then the 3D printed scaffold surfaces were immersed and maintained in contact for ~10 min (as determined to be optimal for surface modification). After removing from the solution, the solvent was allowed to evaporate at (65 °C) to obtain a modified, bioactive surface (Supplementary Figure 7) whilst retaining the bulk properties of the 3D printed scaffolds. (Fig. 3a)

### Material characterisation

Post processed materials were characterised by:

1. Contact angle measurements (Krüss DSA100)-to observe degree of surface wettability
2. FTIR measurements-Jasco FT/IR-4200 spectrometer, Spectra Manager II software.
3. SEM—(Ziess, EVO HD15) to analyse scaffold topography

### Cell culture

Standard cell culture conditions were carried out for both HDF (Life technologies, C-013-5C) and BEC (Caltag Medsystem, SC-3211, SC-3210) as per manufacturer’s advice. 3D printed test scaffolds, which were prepared and transferred to fit within 24-well cell culture plates were immersed in 70% ethanol and rinsed with sterilse PBS before placing the plates with the 3D printed scaffolds under UV radiation overnight in a cell culture hood. The test scaffolds were introduced with respective cell culture media (as noted above) for 3 h at 37 °C to equilibriate and then cells introduced at a cell density of 10,000 cells/well of HDF and at a cell density of 50,000 cells/well of BEC, which were expanded and cultured following supplier instructions under physiological conditions. Cell viability was determined with Alamar blue reagent at 10% (ThermoFisher, DAL1025). Cells adhered on scaffolds were stained with standard protocols with DAPI *ThermoFisher*, D371 and the scaffold was stained with rhodamine before images were obtained using Zeiss Axio Observer Z1.

### Material cell morphology, microscopy and SEM

#### Macro-morphology and pore size estimation

Photographic images of each sample were obtained at 100× magnification with a celestron handheld digital microscope (Celestron, USA) and ImageJ image processing software (National Institute of Health, USA) was used to determine the average size of pores on the respective material surfaces. For scanning electron microscopy, samples were sputter-coated with 5 nm of gold-nanoparticles using a plasma sputter-coater (Quorum Q15ORS) and samples were imaged using JEOL JSM-7401F field emission scanning electron microscope or Zeiss EVO HD15 microscope.

Test samples for SEM with cells were fixed (in 300 μL 0.1 M Sodium Cacodylate buffer with 2% PFA and 1.5% GTA) and critical point dried before being processed for analysis.

### Statistical analysis

*P* < 0.05 was considered statically significant, and the null hypothesis that the data is normally distributed is rejected. Ordinary ANOVA test was conducted for normally distributed data with Bonferroni post hoc test. Kruskal–Walli test was conducted for data that is not normally distributed with Dunn’s post hoc test. Tests samples were kept as *n* = 6 unless otherwise stated.

### Data availability

All relevant data are available from the authors

## Electronic supplementary material

Supplementary Information

Supplementary Figure 1

Supplementary Figure 2

Supplementary Figure 3

Supplementary Figure 4

Supplementary Figure 5

Supplementary Figure 6

Supplementary Figure 7

Supplementary Figure 8

Supplementary Figure 9

Supplementary Figure 10

Supplementary Figure 11

Supplementary Figure 12

Supplementary Figure 13

Supplementary Figure 14

Supplementary Information (to accompany Figure 3C)
