# Supplementary material for: Biomimetic heterogenous elastic tissue development
Source: NPJ Regen Med. 2017 Jun 8;2:16. doi: 10.1038/s41536-017-0021-4 (PMC5678008; doi:10.1038/s41536-017-0021-4)
Supplement: Supplementary file 1 — Supplementary Information [file 41536_2017_21_MOESM1_ESM.docx]

**Supplementary information**

**Supplementary Figures**

**SF 1.**

History and evolution of concepts in repair and regeneration of organs

1. Ancient Greek mythology has referred to natural liver regeneration: an eagle was made to prey on Prometheus' liver, which was renewed as fast as it was devoured. (1611-18; Peter Paul Ruben, Frans Snyders -*Prometheus Bound*; the Philadelphia Museum of Art)
2. A painting of the Saints Cosmas & Damian; patron saints of physicians performing a limb transplant; *The Healing of Justinian,* (The Landesmuseum Württemberg Stuttgart*;* 4th-century) (http://old.bleanchurch.net/historypatronsaints.html)
3. Homunculus (a little man in the round bottom flask) was created by Wagner through alchemy in the fiction, (https://en.wikipedia.org/wiki/Homunculus) *Faust, Part* 2 by Johann Wolfgang von Goethe's, (1832)
4. 600 B.C. text *Sushruta Samhita* details the Indian method of rhinoplasty, which involved cutting skin from the forehead or cheek, thus emphasiing the significance of graft vascularisation.
5. Nobel prize winner, Alexis Carrel described organ perfusion outside of the body that enabled organ transplants ([Science.](http://www.ncbi.nlm.nih.gov/pubmed/17733174) 1935 Jun 21;81(2112):621-3)

**SF 2.**

A filament of TPU80 is entangled in the Cogs of the 3Dison Multi 3D printer, the arrow above point to the cog, which directs the filament into the extruder, the arrow below points to the TPU 80 filament, which is being wraped around the cog instead of being directed down into the extruder head. Finer adjustments (pressure and positioning) to filament guidance

towards the cog and the extruder allowed successful printing with more flexible materials.

**SF 3.**

Chemical structure of the custom made thermoplastic polyurethane used for 3D printing

**SF 4.**

A,B,C,D; Tubular hollow structures were designed in Blender™. An existing mesh structure (cylinder) in Blender™ is modified to include the correct radius and depth, with a modifier to add a thickness to the cylinder, and exported as an STL file into Makerbot slicing software (MakerBot® Industries, LLC, USA) and converted to an .x3g file for printing using an 3Dison Multi 3D printer (Rokit, Korea). The Tubes were printed with either 20, 40, 60 or 80% infill in either a linear or hexagonal pattern using TPU 90 or TPU 80. The structures were printed at a rate of 20mm/s at a temperature of 225ºC.

E,F, G, H, I; *Introducing porous structure within all planes.*

Baisc CAD model of a trachea was modified in +CAD (Simpleware, UK), internal structure wizard to obtain distinct sizes of ‘Schwartz W’ pores with a single infill density. The target volume fraction was set to 60% with 2mm and 3mm pore size. The programme can be used to create ‘pores’ or lattice structures with gradients to mimic varying tissue densities and interphases. The models were converted to an STL file and imported into the makerbot slicing software to be printed by the 3Dison multi. The two models were first printed with Polylactic acid (PLA), a very stable and commonly used filament, to demonstrate the principle. PLA was printed at 230ºC, at a rate of 40mm/s, with the base plate set to 110ºC. The small size created pores of ~772um and the large pores of ~1.28mm

**SF 5.**

1. Comparison of printing definition of TPU80 with TPU 90 using the same codes as used to print TPU 90 (SF 3 A, Linear infill patterns). TPU 80 samples demonstrated defined pores apart from the 80% infill where the larger fibres seemed to coalesce causing the gaps to be filled. the
2. The TPU 80 pores demonstrated no significant difference from the TPU 90 in both 40% and 60% (P>0.05) but were significantly larger in the 20% and significantly smaller in the 80% (P<0.001).

**SF 6.**

3D printed TPU 90 scaffolds (15 x 15 x 1 mm) to have a rectilinear pattern with corresponding infill density being subjected to biaxial forces.

**SF 7.**

FTIR analysis of a) 3D printed scaffolds TPU before and after biofunctionalisation with b) pre-polymer mixed c) Collagen and d) L-AME (before L-AME was removed)

**SF 8.**

Alamar blue assay for cell viability tests. Surface functionalized scaffolds have significantly higher cell adhesion (at 24hr with HDF, and at 72hr with BEC) compared to control scaffolds without surface treatments. However prolong cell culture (at 72hr with HDF and at 14 days with BEC) demonstrated that the scaffolds, which were not surface treated can equally allow cell survival as surface functionalized scaffolds with no significant difference in cell viability. All values are signifianctly different (*p<0.05) except L-AME+Collagen between the test time points (between 24hr and 72hr with HDF and between 72hr and 14days with BEC)

**SF 9.**

1. Step by step process which was followed for designing a tracheal construct with Blender™ to obtain an stl file
2. Stl of the tracheal construct designed on Blender™ is processed in Slic3r™ for dual printing; tracheal ‘rings’ with TPU 90 and the ‘body’ with TPU 80. Indicating in yellow is the 3 layers of perimeter on each of the constructs, and indicated in red is the infill with 10% ‘honeycomb’ pattern. A G-code was obtained to 3D print this structure.(figure 3)

**SF 10.**

3D printed TPU tracheal construct was anastomosed with anchored sutures in an interrupted style (4.0 proline, ethicon llc, Johnson and Johnson, USA) to a porcine trachea with the lungs intact. The system was placed in a 0.9% NaCl bath and introduced with air through the pressure bag to mechanically inflate/deflate the lungs to confirm airtightness of the 3D printed tracheal structure and ease of suturing to the scaffold.

Porcine tissues were harvested through a tissue sharing scheme operated through the Royal Veterinary College, University of London. The experiments in which the animals were involved were carried out under the ethical approval provided by the College’s Ethics and Welfare Committee (animals were not killed for the specific purpose of the experiments carried out in this study)

**SF 11.**

A. dimensions of the 50mm Dumbbell samples used for tensile tests. B, D, F,H,J, L indicate distinct locations on the TPU structure where dumbbells were extracted for tension testing. B, C longitudinal segments of the anterior wall. D, E longitudinal dumbbell samples at the junction between anterior and posterior wall. F, G indicate radial dumbbell samples of the posterior wall. H, I indicate longitudinal Dumbbells of the posterior wall. J, K, Radial dumbbell samples of the Cartilage ring mimics. L radial dumbbell samples between the cartilage rings. M, a dumbbell sample held between the grips of Instron to determine tensile properties. B, C, J, L represent the anterior wall and D, E, F, G, H, I represent the posterior wall.

**SF 12**.

Compliance measurements were set up for tracheal construct testing. The ultrasound probe is resting above, across the TPU trachea, which is connected on either ends to endotracheal cuff inflators.

**SF 13.**

Generic tracheal structure was designed with CAD but with distortion to the physiological dimensions (17/26) instead (25/23) to investigate corresponding difference in mechanical properties with subtle changes to the basic design. Force required for anterior compression was nearly three times greater than in the lateral direction (p<0.001)

**SF 14.**

Filament formation. 1.75mm filaments were extruded using a standard plastic extrusion set up.
