# Supplementary figures and images for: Biomimetic heterogenous elastic tissue development
Source: NPJ Regen Med. 2017 Jun 8;2:16. doi: 10.1038/s41536-017-0021-4 (PMC5678008; doi:10.1038/s41536-017-0021-4)

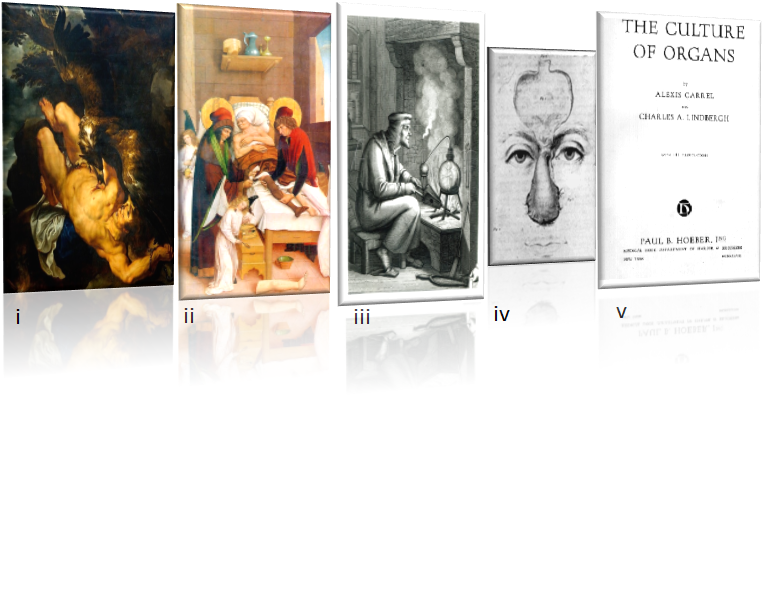

Supplement: Supplementary file 2 — Supplementary Figure 1 [file 41536_2017_21_MOESM2_ESM.png]

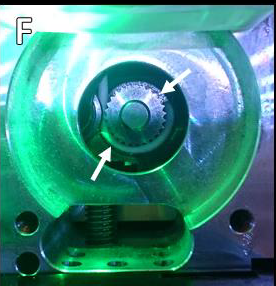

Supplement: Supplementary file 3 — Supplementary Figure 2 [file 41536_2017_21_MOESM3_ESM.png]

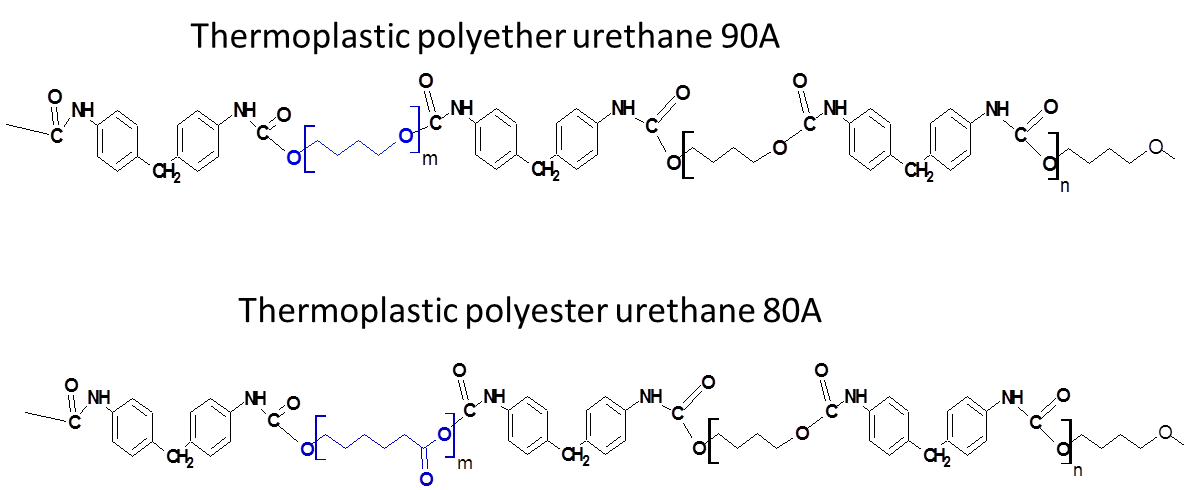

Supplement: Supplementary file 4 — Supplementary Figure 3 [file 41536_2017_21_MOESM4_ESM.png]

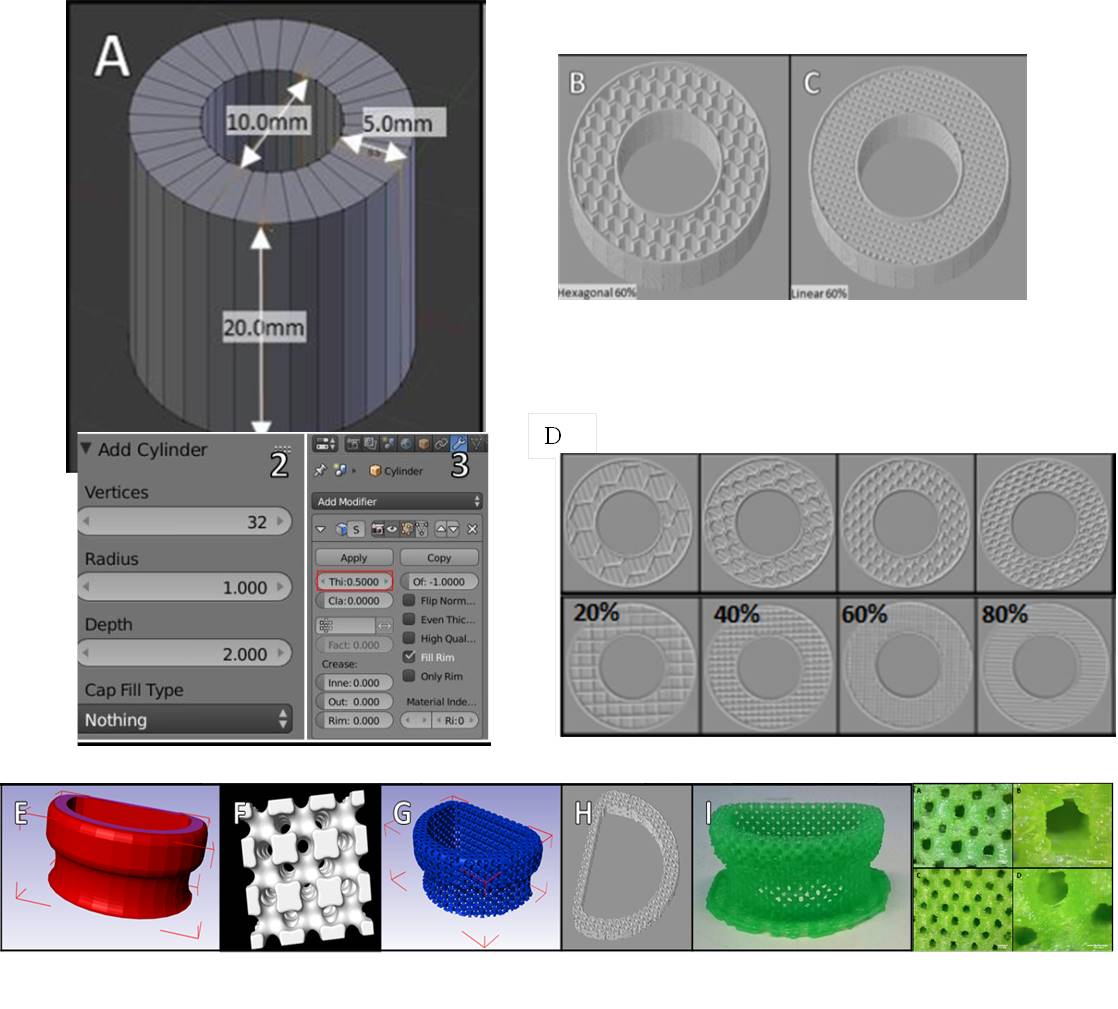

Supplement: Supplementary file 5 — Supplementary Figure 4 [file 41536_2017_21_MOESM5_ESM.jpg]

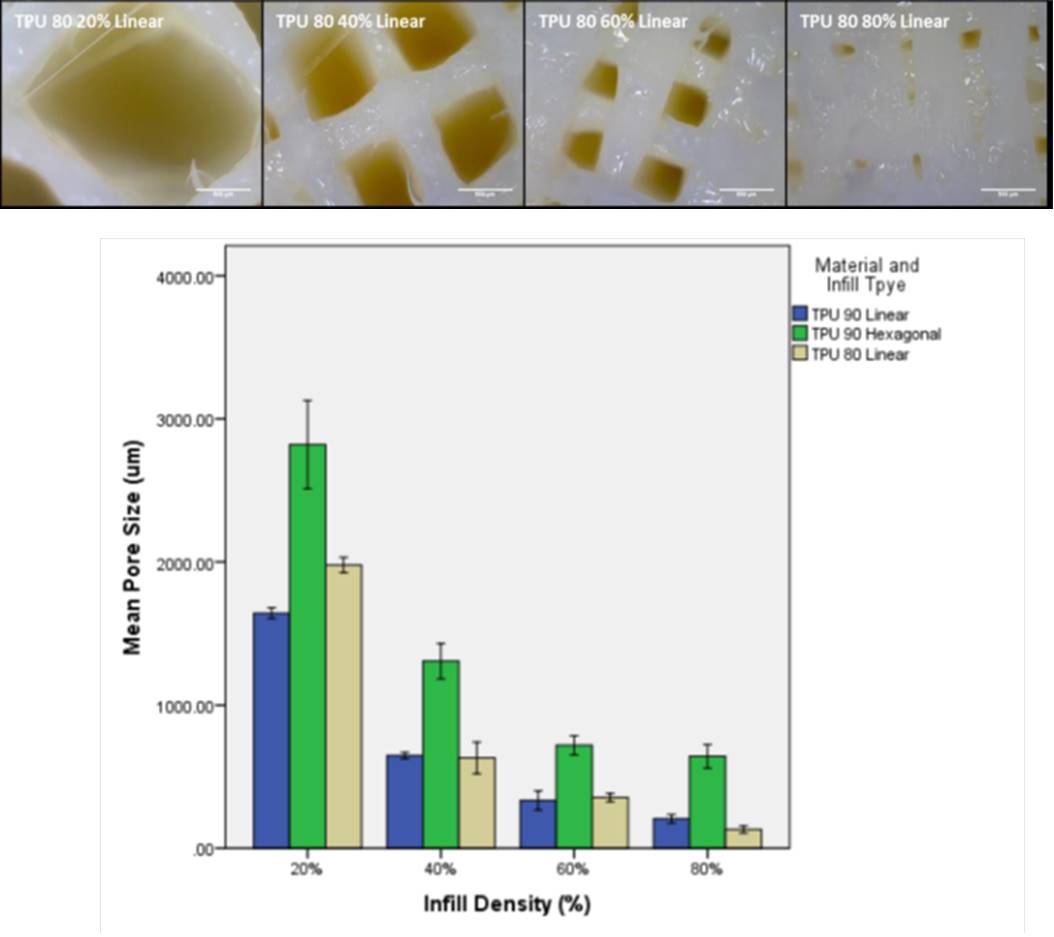

Supplement: Supplementary file 6 — Supplementary Figure 5 [file 41536_2017_21_MOESM6_ESM.jpg]

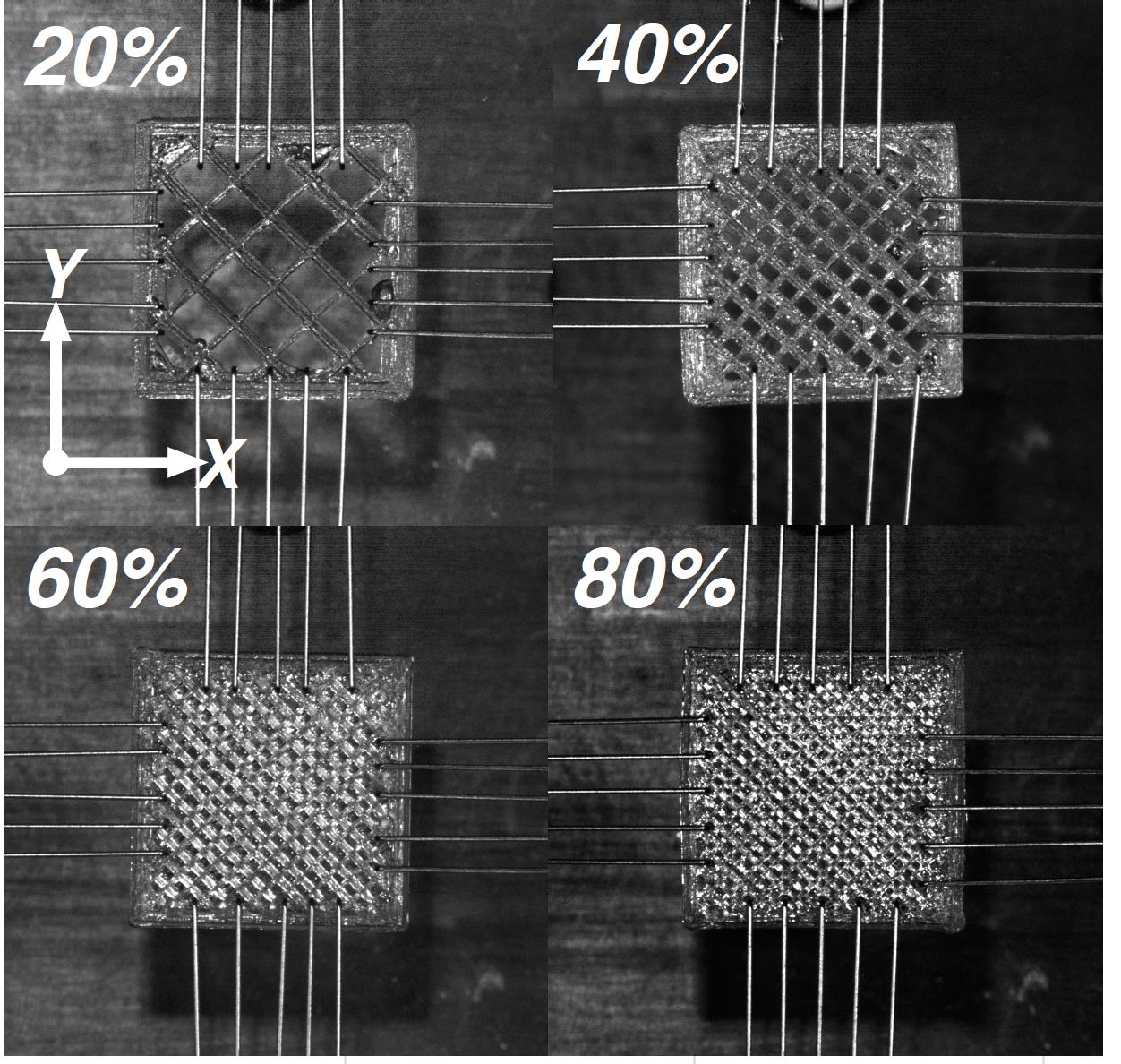

Supplement: Supplementary file 7 — Supplementary Figure 6 [file 41536_2017_21_MOESM7_ESM.jpg]

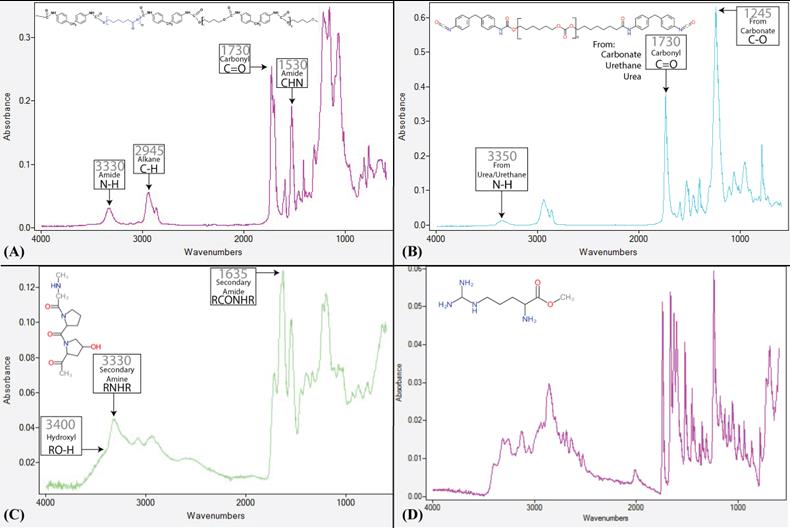

Supplement: Supplementary file 8 — Supplementary Figure 7 [file 41536_2017_21_MOESM8_ESM.jpg]

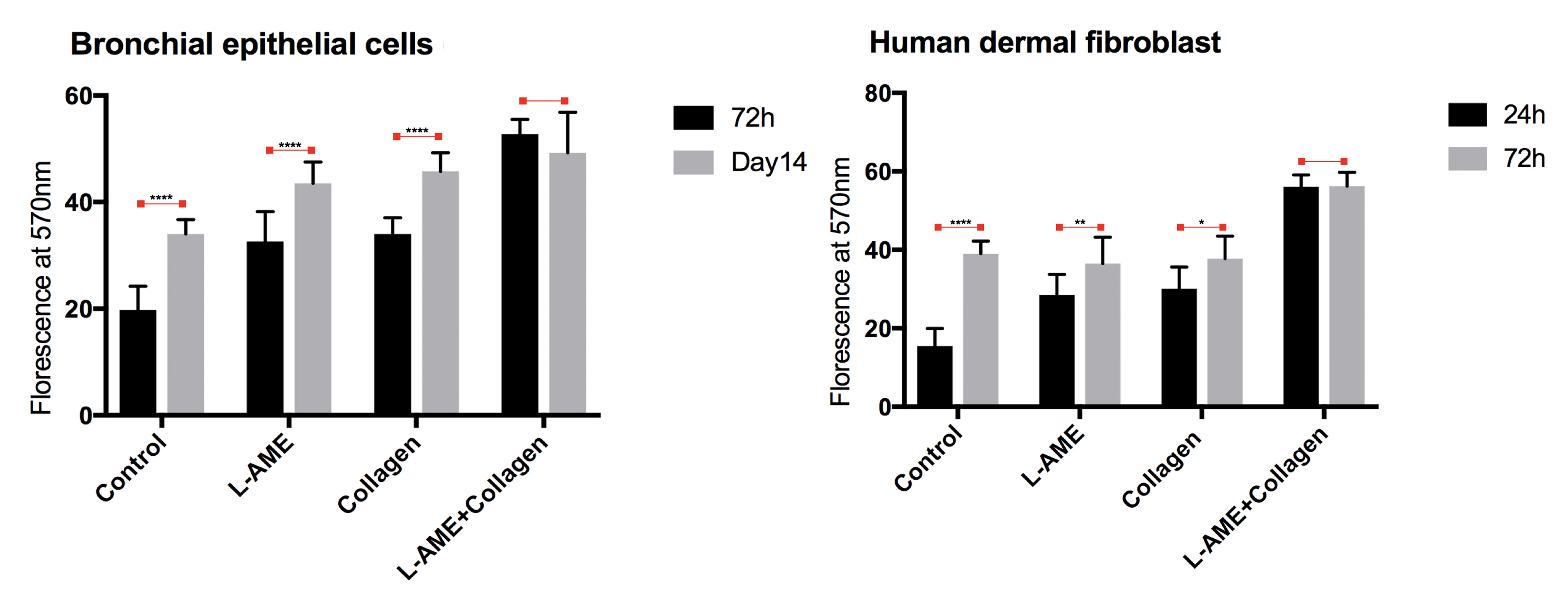

Supplement: Supplementary file 9 — Supplementary Figure 8 [file 41536_2017_21_MOESM9_ESM.jpg]

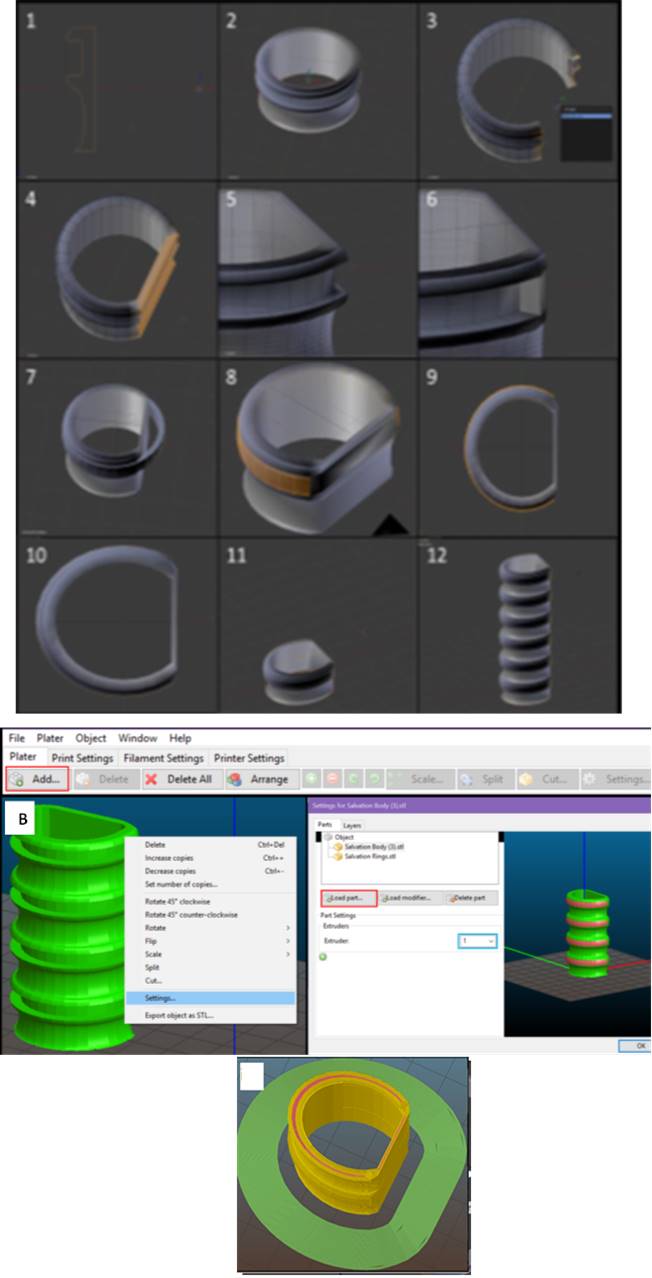

Supplement: Supplementary file 10 — Supplementary Figure 9 [file 41536_2017_21_MOESM10_ESM.jpg]

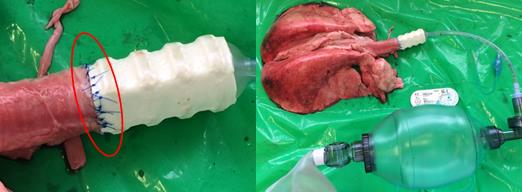

Supplement: Supplementary file 11 — Supplementary Figure 10 [file 41536_2017_21_MOESM11_ESM.jpg]

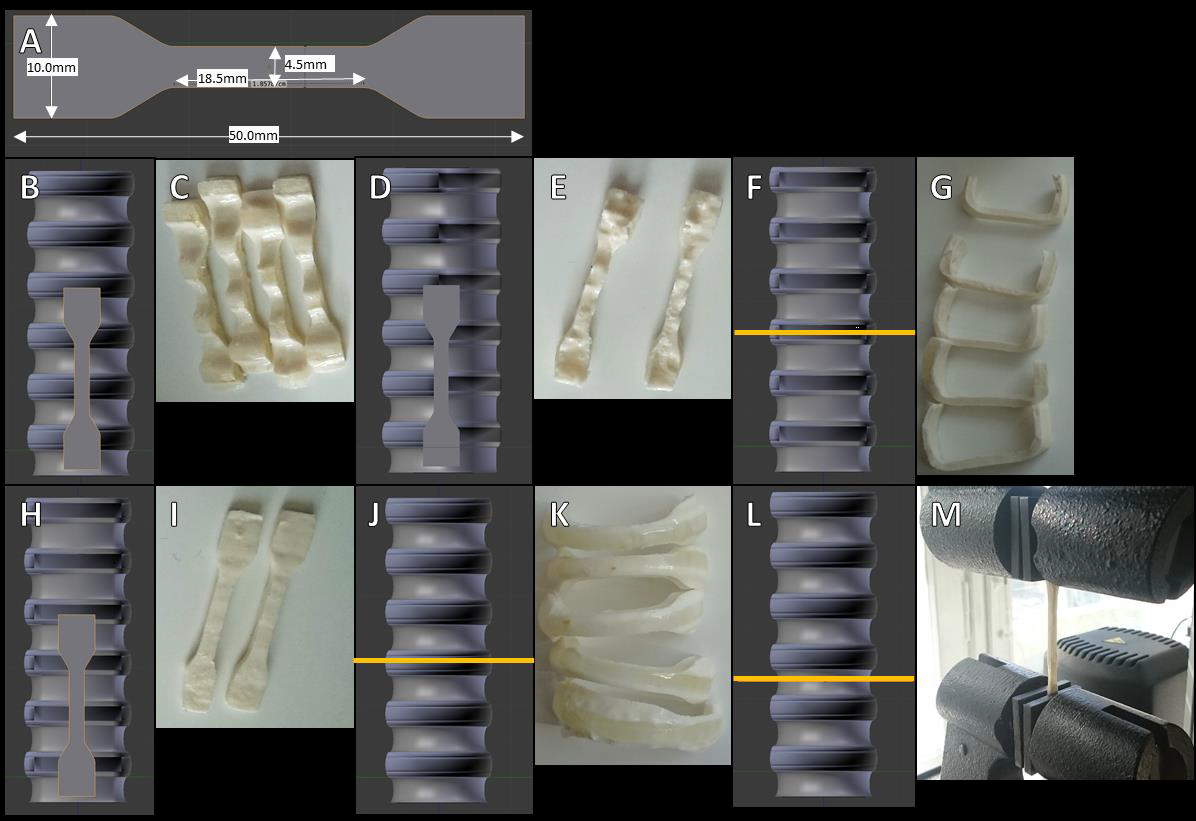

Supplement: Supplementary file 12 — Supplementary Figure 11 [file 41536_2017_21_MOESM12_ESM.png]

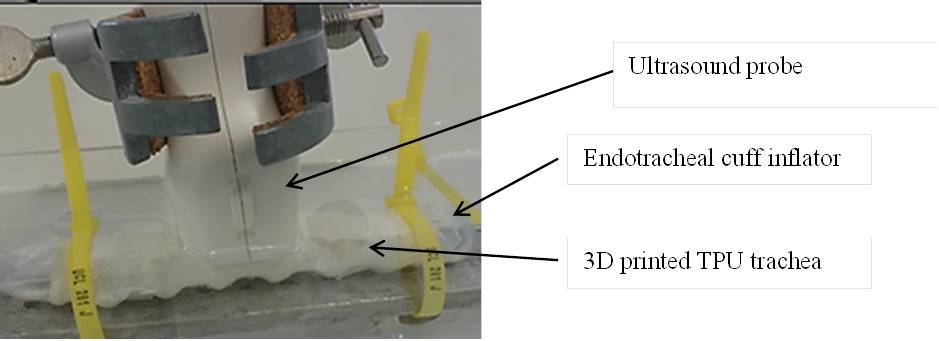

Supplement: Supplementary file 13 — Supplementary Figure 12 [file 41536_2017_21_MOESM13_ESM.jpg]

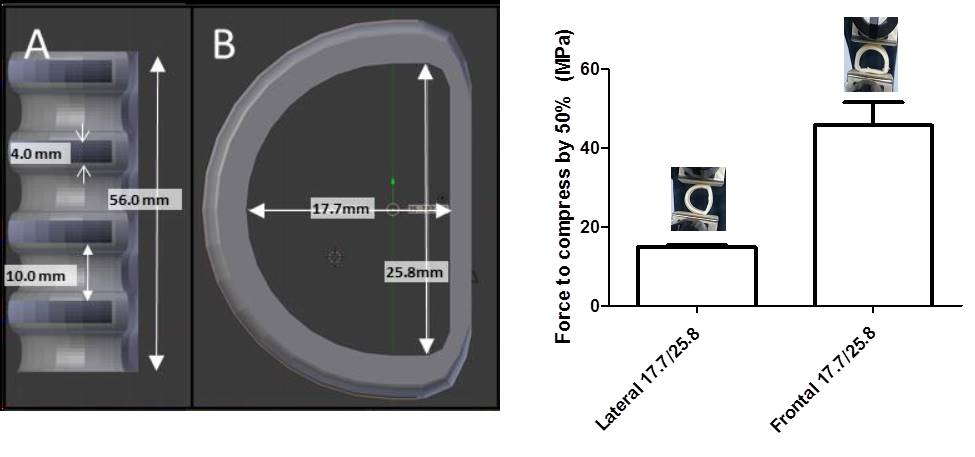

Supplement: Supplementary file 14 — Supplementary Figure 13 [file 41536_2017_21_MOESM14_ESM.jpg]

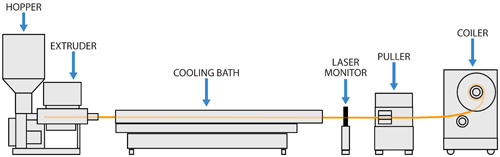

Supplement: Supplementary file 15 — Supplementary Figure 14 [file 41536_2017_21_MOESM15_ESM.jpg]

## Slide 1
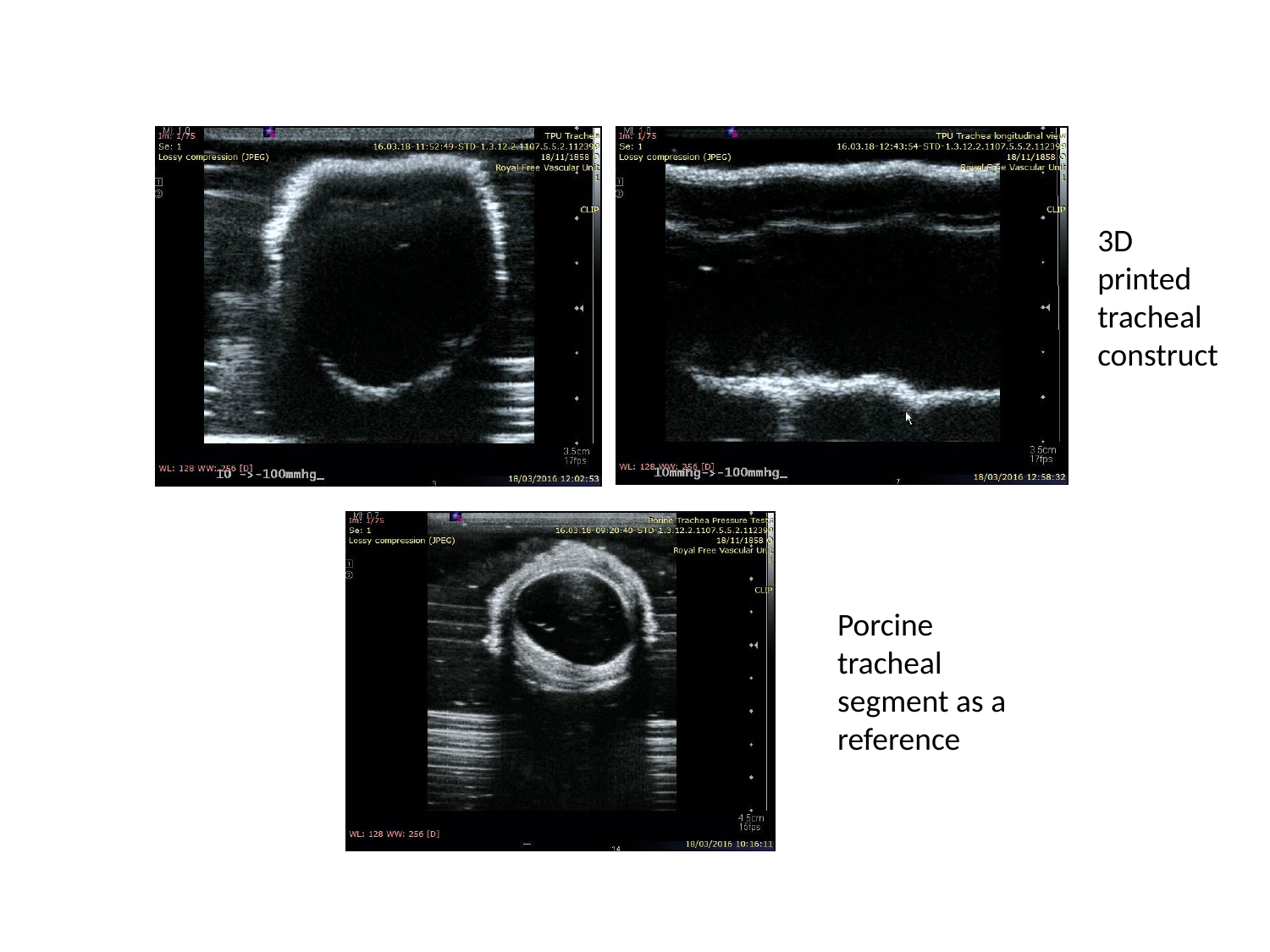

3D printed tracheal construct
Porcine tracheal segment as a reference

Supplement: Supplementary file 16 — Supplementary Information (to accompany Figure 3C) [file 41536_2017_21_MOESM16_ESM.pptx]
